# Immunostaining study of cytokeratins in human hair follicle development^☆^^☆☆^

**DOI:** 10.1016/j.abd.2019.09.028

**Published:** 2020-03-21

**Authors:** Laura Maria Andrade Silva, Ricardo Hsieh, Silvia Vanessa Lourenço, Neusa Yuriko Sakai Valente, Geise Rezende Paiva, Juliana Dumet Fernandes

**Affiliations:** a Graduate Program in Medicine and Health, Faculty of Medicine of Bahia, Universidade Federal da Bahia, Salvador, BA, Brazil; b Service of Dermatology, Escola Bahiana de Medicina e Saúde Pública, Salvador, BA, Brazil; c Service of Dermatology, Hospital Santa Izabel, Salvador, BA, Brazil; d Department of Dermatology, Faculdade de Medicina, Universidade Federal da Bahia, Salvador, BA, Brazil; e Instituto de Medicina Tropical de São Paulo, Universidade de São Paulo, São Paulo, SP, Brazil; f Department of Pathology, Universidade Federal de Alfenas, Alfenas, MG, Brazil; g Faculdade de Odontologia, Universidade de São Paulo, São Paulo, SP, Brazil; h Department of Dermatology, Faculdade de Medicina, Hospital das Clínicas, Universidade de São Paulo, São Paulo, SP, Brazil; i Sector of Pathology, Hospital Santa Izabel, Escola Bahiana de Medicina e Saúde Pública, Salvador, BA, Brazil

**Keywords:** Hair follicle, Hair-specific, Keratins, Molecular, Pathology

## Abstract

**Background:**

The hair follicle is a unique structure, one of the most dynamic structures in mammalians, which can reproduce in every new cycle all the mechanism involved in its fetal development. Although a lot of research has been made about the human hair follicle much less has been discovered about the importance of the cytokeratins (CKs) in its development.

**Objective:**

Study the immunohistochemical pattern of epithelial CKs during human hair follicle development.

**Methods:**

We performed an immunohistochemical study using fresh post-mortem skin biopsies of human fetuses between 4 and 25 weeks of gestational age to study the expression of cytokeratins (CKs): CK1, CK10, CK13, CK14, CK16 and CK20 during human hair follicle fetal development.

**Study limitations:**

Restrospective study with a good number of makers but with a small population.

**Results/conclusion:**

We found that, the CKs were expressed in an intermediate time during follicular development. The epithelial CKs (CK1, CK14, CK10, CK13) and the epithelial CKs with a proliferative character such as CK16 were expressed first, as markers of cellular maturation and follicular keratinization. At a later phase, CK20 was expressed in more developed primitive hair follicles as previously discussed in literature.

## Introduction

The hair follicle is in constant renewal, reproducing the same embryonic mechanisms that formed it first in the fetal period.^1^ The hair follicles develop at early phases of follicular development, around the 9th and 12th week originating from a germinative stratum of the epidermis's proliferation. The keratinocytes differentiate and form the outer root sheath (ORS), the companion sheath, the inner root sheath, and the hair shaft itself.^2^ Its development respects a cranial caudal pattern of development with the first hair follicles being observed at face and eyebrow.^2^

An extremely challenging area is how follicular development and its maturation occurs, follicular development and its subsequent differentiation involves a complex series of events that generate cell matrix remodeling, molecules such as proteoglycans and growth factors are implicated in this process.^3^, ^4^, ^5^, ^6^

Cytokeratins (CKs) are the main proteins that structure epithelial cells, they provide shape, resistance and maintenance of the intercellular contacts; in the hair follicle in addition to the structural function, they are markers of follicular maturation and cytodifferentiation. They are divided into two families, Type 1, acid keratins and Type 2, basic keratins.^7^ In total, twenty-six keratins are considered specific to hair follicle, comparable to the twenty-nine expressed in the epithelium.^8^

A range of mutations in CKs are related to diseases that affect hair structure and density in humans and animals. Diseases such as hypotrichosis and monilethrix have already been linked to specific mutations of some CKs.^9^, ^10^, ^11^ Other diseases that may affect the hair follicle as lichen planus pilaris show an altered pattern in the expression of certain CKs that could be used as early markers for this disease.^12^, ^13^ Thus, CKs may be targets for drug development and diagnostic methods that may assist in the early diagnosis and treatment of hair follicle diseases.

Although there is already a vast literature on hair follicles and epithelial CK, little is known about the expression of epithelial CK during the differentiation of human hair follicles. Considering the importance of CK in the development of hair follicles and the scarcity of information about this subject, we studied the pattern of epithelial CK expression in the developing human hair follicle.

## Methods

A retrospective study involving histological sections from hairy areas from skin fragments of human embryonic/fetal derivatives from different gestational ages (4–25 weeks) were obtained from the archives of the Dermatopathology Laboratory, Department of Dermatology, University of São Paulo. The study submitted to the Ethics Committee of this institution. CEP no. 0172/08. The analyzed regions were the same and standardized in the groups as: scalp, face, eyebrow, upper limb, back and genitalia. The morphologies of the structures were previously evaluated in hematoxylin and eosin for better visualization of the structures. In these sections, an immunohistochemical study was performed with the following markers: CK1, CK10, CK13, CK14, CK16 and CK20.

### Immunohistochemical protocol

The 3 μm sections were dewaxed and diaphanized in two xylol baths. The specimens were then rehydrated in ethanol drop-off and immersed in 10% ammonium hydroxide solution. Recovery of the antigenic sites occurred in various ways depending on the primary antibody used (Table 1).

**Table 1:** Monoclonal antibodies used in study and protocols.

Primary anti body	Clone	Company	Antigenic retrieval	Dilution	Incubation
CK1	34BB4	Novo Castra	Microwave	1:25	“Overnight”
CK14	AB-1	Neomakers	Microwave	1:40	“Overnight”
CK16	DE-K10	Dako	Microwave	1:50	“Overnight”
CK10	DE-K10	Dako	Microwave	1:50	“Overnight”
CK13	M 7003	Dako	Microwave	1:50	“Overnight”
CK20	Ks20.8	Dako	Microwave	1:50	“Overnight”

After washing, the material was incubated in 20 volumes hydrogen peroxide solution, and then the specimens were immersed twice in TRIS pH 7.4 solution and subsequently incubated with 0.5% BSA (Sigma) solution, 2.5% SFB (Cultilab) in TRIS and incubated with the primary serum diluted in 1% BSA (Sigma) solution in TRIS. Subsequent procedures were always preceded by washes in the TRIS buffer. After incubation with the primary antibody, the specimens were incubated with En Vision reagent (Dako). For the development, the specimens were incubated in the solution containing DAB agent (Sigma). The histological sections were then counterstained with Carazzi Hematoxylin and subsequently dehydrated in upstream alcohols, diaphanized in three xylol baths, and mounted on Permount® resin for microscopic examination. Negative controls were performed by incubating specimens with non-immune serum. All immunohistochemical reactions were performed in parallel with adult tissues as negative/positive controls.

The results obtained in the immunohistochemical reactions were analyzed by two observers and qualitatively tabulated. The specimens that showed immunostaining were considered positive (+), the specimens that did not present immunoexpression were considered negative (−).

All the results were recorded by the Axio Vision digital photomicrograph system (Zeiss and Axiophot).

## Results

**Figure 1:**
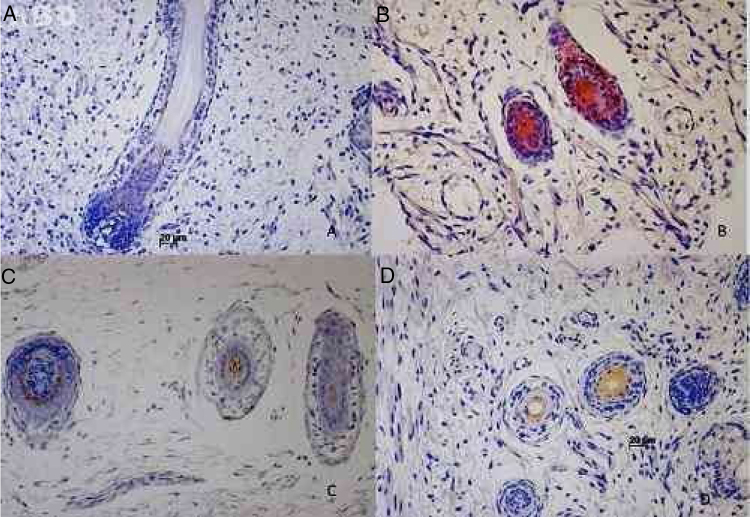
Cytokeratin's immunostaining in human hair follicle development. (A) CK1 immunoexpression at 19 weeks fetus. (B) CK14 immunoexpression at 12 weeks fetus. (C) CK20 immunoexpression at 21 weeks fetus. (D) CK16 immunoexpression at 12 weeks fetus.

73 specimens from hairy areas of embryos or fetuses ranging from 4 to 25 weeks of gestational age were evaluated. The following markers were evaluated: CK1, CK10, CK13, CK14, CK16 and CK20, in the regions such as: scalp, face, eyebrow, upper limb, back and genitals and the results can be evaluated in Table 2 and Fig. 1. We analyzed all the cases obtained in the files of the Dermatopathology Laboratory referring to hairy areas of each marker. A total of 12 CK1 specimens, 14 CK14 specimens, 9 CK10 specimens, 10 CK13 specimens, 21 CK16 specimens and only 7 CK20 specimens were obtained.

**Table 2:** Resume of cytokeratins's immunoexpression at human hair follicle development.

Marker	Immunostannig percetual	First gestational age immunoexpression
CK1	25%	12 weeks
CK14	100%	9 weeks
CK10	55%	12 weeks
CK13	70%	12 weeks
CK16	61%	12 weeks
CK20	57%	14 weeks

### CK1, CK14, CK10 and CK13

Immunostaining for CK1, CK10, CK13 was noted from the 12th week gestation on the primitive hair follicles and up to the 25th gestational week on the most fully developed follicles. 25%, 55%, 70% of the specimens marked positively for CK1, CK10 and CK13 respectively, in ORS, in the territory of the follicular ostium, infundibulum, sebaceous gland and dermal papilla. The main marked regions were the eyelid, face and genital. Immunostaining for CK14 was seen in primitive follicles of 9 week gestation embryos in the upper limb and back, and even in more developed follicles at 21 gestational weeks. 100% of the cases marked positively for CK14 in ORS. All regions analyzed were positively marked.

### CK16 and CK20

Immunostaining for CK16 was seen in primitive follicles of 12 week gestation embryos in the facial region and in more developed 23 week gestational follicles. 61% of the specimens marked positively for CK16 at ORS. All regions analyzed were marked positively. Immunostaining for CK20 was seen from primitive follicles of 14 week gestation embryos in the scalp region to more developed 25 week gestational follicles. 57% fetuses marked for CK20 in the territory of ORS and dermal papilla. All regions analyzed were marked.

## Discussion

This study evaluated the pattern of epithelial CK expression in developing human hair follicle. There are few papers in the literature about this subject.

Schirren et al. were the pioneers showing that the follicular cells of the human fetal placodes expressed CK5, CK6, CK14, CK17 and CK19, and that subsequently with follicular development the pattern of expression of those CK changes in the each portion of the hair follicle.^14^

Our data showed that between 9 and 14 gestational weeks, CK1, CK14, CK10, CK13, CK16 and CK20 became expressed. Only two of our markers were evaluated by Schirren et al., CK14 and CK10, these CKs were found in the developing hair follicles, CK14 in the earliest phases still in the placoid phase and at later phases, CK10 was express in a slightly more differentiated hair follicle in the isthmus region in ORS at the supra basal cells of infundibulum along with CK14. The results of that work were similar to ours; the immunostaining maintained the cranial pattern of distribution.

In our study, CK14 was the first CK to be expressed in embryos with less than 12 weeks of life, around the 9th gestational week. Initially, both CK1 and CK14 were expressed in the region on the isthmus. In 2008, Lourenço et al. when analyzed the expression pattern of CKs in the primitive fetal epidermis, showed CK1 expression was noted in all epidermal layers while CK14 was noted predominated in the perifollicular epidermal region.^15^

In our study, CK1, CK10, CK13 and CK16 were expressed at 12 weeks of gestational age. Studies involving CK1 and CK14 at Shh signaling pathway have shown conflicting findings. When occurs the overexpression of CK1 at mices in embryonic period, they were unable to develop hair follicles. In the other experimental model, when occurs the superexpression of CK14, a follicular hyperproliferation occurred, in addition to multiple lesions such basal cell carcinoma in the animals analyzed.^16^, ^17^

It is reported in the literature that hyperproliferation of epithelial layers caused by overexpression of CK1 during embryonic development alters the ability of basal cells to initiate a suitable cell differentiation program for hair follicle formation.^16^ Thus, CK1 and CK14 plays an important role in the formation of the fetal hair follicle through the Shh signaling cascade. The exact time, location, and concentration of expression of these Cks in this Shh signaling pathway affect the hair follicle phenotype in the embryonic fetal period, but without changes in the postnatal hair follicles.^16^

CK10 is an epithelial CK classically related to Epidermolytic hyperkeratosis, eventual mutations may be related to this disease or similar ones,^18^ in our work it was seen marking the hair follicles in ORS, as well as CK1 and CK14.

In our results, CK16 was expressed in more proliferative regions of hair follicle, mainly in the bulb region. CK16, as well as CK6 and CK17 are proliferative and repairing CKs, being expressed during diseases and around healing tissues. Corroborating with our findings, it is described that these hyperproliferative CKs are expressed in the hair follicles at an intermediate stage of development, when epithelial differentiation already exists.^14^

CK13 is an epithelial CK already used as an immunotarget agent for oral intraepithelial neoplasia, more recently its expression with CK17 has been evaluated as an immunotarget for vulvar intraepithelial neoplasia as well. Our work evaluated CK13 at the developing hair follicle and saw it marking fetal hair follicles's ORS as well as the other epithelial CKs.^19^

CK20 is a marker for Merkel cells. As in the literature, its immunoexpression was detected in hair follicles at a later stage of development, from the 14th week of gestational age. The literature reports the presence of Merkel cells in the ORS of hair follicles of rodent vibrissae, in humans CK20 is reported to mark at the basal layer of the infundibulum at primitive fetal hair follicles, not marking at deep areas of the hair follicles, including the bulb and the dermis.^20^ In our work, according to the literature, we found expression for CK20 in the hair follicles already developed around the 25th gestational week in the dermal papilla and in the ORS, but also in the follicles in the intermediate period of development with 14 gestational weeks.

## Conclusion

Hair follicle morphogenesis is a restructuring of epithelial tissue and mesenchymal tissue to form a completely differentiated pilosebaceous unit. We have observed that CK expression patterns during hair follicle development are as dynamic as seen by other authors in the epidermis.^15^ CKs begin their expression in human hair follicles around the 9th gestational week, with CK14 being the first to be identified and with the highest percentage of immunostaining. Our data confirm the literature showing that CKs are markers of maturation of hair follicle development and are expressed from the follicular placoid phase. Initially the expression of these CKs coincides with the CKs expressed in the primitive epithelium: CK1, CK14, CK10, CK13 and proliferative CK: CK16. As previously reported in the literature, CK20 was expressed in more developed hair follicles from the 14th gestational week. The results when taken together indicate that from the earliest stages of follicular development the hair follicles progressively and coordinately prepare to protect the fetal skin for birth and environmental stimulation. Understanding of these maturation steps is important for understanding the structure of normal hair follicles and their adult function in health and disease. Our work has served to fill a gap in the literature on a poorly studied topic. We also believe that by identifying patterns of differentiation of CKs in hair follicles in humans, we can raise questions and prompt investigations, and open the possibility for regenerative therapies, identification of early biomarkers and potential diagnostic targets for hair follicle diseases. However, further investigative studies are needed to better evaluate the development and protein expression of human hair follicles and to collaborate in the diagnosis and treatment of diseases related to this structure.

## Financial support

FUNADERM – Fundo de Apoio à dermatologia Fundação de Apoio ao dermatologista (CAPES - Coordenação de Aperfeiçoamento de Pessoal de Nível Superior).

## Authors’ contributions

Laura Maria Andrade Silva: Statistic analysis; approval of the final version of the manuscript; elaboration and writing of the manuscript; obtaining, analysis, and interpretation of the data; effective participation in research orientation; intellectual participation in the propaedeutic and/or therapeutic conduct of the studied cases; critical review of the literature; critical review of the manuscript.

Ricardo Hsieh: Approval of the final version of the manuscript; conception and planning of the study; effective participation in research orientation; critical review of the manuscript.

Silvia Vanessa Lourenço: Conception and planning of the study; effective participation in research orientation; intellectual participation in the propaedeutic and/or therapeutic conduct of the studied cases; critical review of the manuscript.

Neusa Yuriko Sakai Valente: Approval of the final version of the manuscript; conception and planning of the study; effective participation in research orientation; critical review of the manuscript.

Geise Rezende Paiva: Approval of the final version of the manuscript; obtaining, analysis, and interpretation of the data; critical review of the manuscript.

Juliana Dumet Fernandes: Approval of the final version of the manuscript; conception and planning of the study; elaboration and writing of the manuscript; obtaining, analysis, and interpretation of the data; intellectual participation in the propaedeutic and/or therapeutic conduct of the studied cases; critical review of the literature; critical review of the manuscript.

## Conflicts of interest

None declared.
